# A Non-Conventional Review on Multi-Modality-Based Medical Image Fusion

**DOI:** 10.3390/diagnostics13050820

**Published:** 2023-02-21

**Authors:** Manoj Diwakar, Prabhishek Singh, Vinayakumar Ravi, Ankur Maurya

**Affiliations:** 1 Department of CSE, Graphic Era Deemed to be University, Dehradun 248002, India; 2 School of Computer Science Engineering and Technology, Bennett University, Greater Noida 201310, India; 3 Center for Artificial Intelligence, Prince Mohammad Bin Fahd University, Khobar 34754, Saudi Arabia

**Keywords:** multi-modality, wavelet transform, image fusion, edge detection, texture detection

## Abstract

Today, medical images play a crucial role in obtaining relevant medical information for clinical purposes. However, the quality of medical images must be analyzed and improved. Various factors affect the quality of medical images at the time of medical image reconstruction. To obtain the most clinically relevant information, multi-modality-based image fusion is beneficial. Nevertheless, numerous multi-modality-based image fusion techniques are present in the literature. Each method has its assumptions, merits, and barriers. This paper critically analyses some sizable non-conventional work within multi-modality-based image fusion. Often, researchers seek help in apprehending multi-modality-based image fusion and choosing an appropriate multi-modality-based image fusion approach; this is unique to their cause. Hence, this paper briefly introduces multi-modality-based image fusion and non-conventional methods of multi-modality-based image fusion. This paper also signifies the merits and downsides of multi-modality-based image fusion.

## 1. Introduction

There is currently a wide range of image-processing techniques available to generate optimal imaging quality for diagnostic purposes. The best quality image is crucial to gaining good visual information. Moreover, the fundamental strategy for image processing converts an analog image into a digital image. It performs some operations and calculates the mathematical form by using a type of signal processing with an image as input and a series of all images on it as output [1,2,3,4,5].

Various kinds of medical images are used to distinguish applications such as CT, PET, and MR images. The pixel is a crucial part of any image, and this little picture element has some coordinates and intensity color values [6,7,8,9,10]. The various digital image examples [11,12,13,14,15,16,17] represent images performed in relevant space and time by sampling. It is crucial to use a few processing operations block-wise, and the pixel-to-pixel operation on the image is the primary operation through which we can resolve the issue of some pixels overlapping [18,19,20,21,22].

Signal distribution or characteristics are associated with image processing operations to extract better image quality and some significant information. The set for f (x, y), where x and y are spatial coordinates and the amplitude of any pair of coordinates, can be used to determine two-dimensional (2D) images. The digital signal-processing operations are performed on the digital images [23,24,25,26,27,28]. The several operations performed are the enhancement of the image, the restoration of the image, the compression of the image, and the segmentation of the image [29,30,31,32,33]. These operations perform and concentrate on image enhancement to improve image quality during image processing. Image fusion is the merging of complementary information about two or more images into a single output image. Image fusion is widely used in several applications related to remote sensing, medical imaging, the military, and astronomy [34,35,36,37,38,39,40]. Image fusion is the technique of combining images to enhance the content information in the images. Image fusion methods are critical for improving the performance of object recognition systems by combining many different sources of images taken from different satellite images, and airborne images, and relying on ground-based systems for the different datasets [41,42,43,44,45]. The advantages, disadvantages, and applications of the fusion process are discussed in Table 1.

### Major Contributions

Some of the most important contributions of this non-conventional multi-modal medical image fusion survey are listed below:

1. A detailed introduction to non-conventional multi-modal medical image fusion techniques is presented. Most of the works selected for this survey are recent;
2. In addition, an analysis of non-conventional strategies for fusing many types of medical images is performed. Using multi-modal-source images generated from a CT scan, a SPECT, an MR-T1 image, and an MR-T2 image, six typical medical image fusion algorithms are compared and contrasted based on the results of five prominent objective metrics;
3. Some future research potentials for non-conventional multi-modal image fusion are proposed, while the existing difficulties in this area are highlighted.

The rest of this paper is organized as follows: Section 2 presents a brief introduction to the background of the techniques used in multi-modality image fusion. Section 3 is about the related work of medical image fusion. A comparative analysis of non-traditional related work has been critically discussed in Section 4. The outcomes with visual analysis and performance metrics are discussed in Section 5. Section 6 concludes the paper with a future perspective.

## 2. Multi-Modality Image Fusion

Multi-modality image fusion entails a composition of the image taken from different medical sources and equipment to acquire more detailed and reliable information about the image. In recent trends, radiography synthesis has used multi-modality in medical diagnosis and treatment. These methods of cure are adopted for diagnosing or excluding the disease. Medical images are classified into several categories; they can be distinguished in the image based on the various human body functions and physical structure of the image, which has a relatively low functional image spatial resolution. Thus, it can provide information about blood circulation and visceral metabolic rate. As in Table 2, MR and CT image fusion CT images show the physical details, while MR images show the functional details.

**Figure 1:**
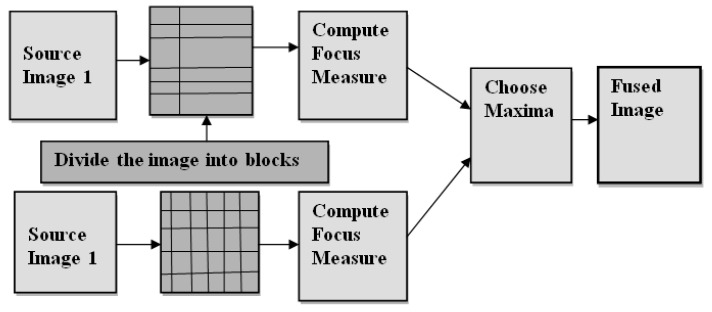
An illustration of the image fusion method using block-wise focus measure rule in the spatial domain.

**Figure 2:**
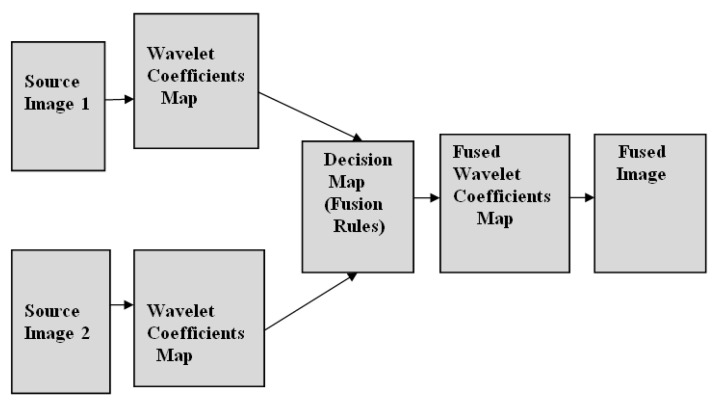
An illustration of the image fusion method using decision map rule in the wavelet domain.

In multimodality medical image fusion, the rules are applied in the spatial domain and wavelet domain. Figure 1 shows an illustration of the image fusion method using the block-wise focus measure rule in the spatial domain. In this block, both source images are divided, and by computing the focus measure and choosing the maxima rule, a fused image is obtained. Similarly, Figure 2 shows the image fusion method using the decision map rule in the wavelet domain. Here, image fusion is performed in the wavelet domain by decomposing the approximation and detail parts.

In a recent area of research, medical imaging that provides a representation of the object plays a crucial role in the field of medical treatment [46,47,48]. The complete structure of the spectrum in digital image processing is helpful in medical diagnosis. For good treatment, radiologists have to combine organs or diseases. Moreover, because of design constraints, instruments cannot provide this type of information. For the superior quality of the image, distinguishing conditions in image processing demand high spatial and spectral information in a single image [49,50,51,52,53]. In the medical field, the significance of medical images is distinguished from other images. The significance of body organs or living tissues present in medical images can be correctly analyzed by improving the heterogeneous areas of the images. The objects obtained with identical modality and size may vary from one patient to another; they are defined through a standardized acquisition protocol in terms of shape, internal structure, and sometimes various views of the identical patient at identical times [54,55]. In biological anatomy, object delineation cannot be erased from the background. Automatic image analysis in the field of medicine does not provide fake measurements. Rather, the robustness of the algorithm does: because those images cannot be handled properly, they are simply rejected. This illustration shows that image fusion enhances the quality of the image. In multimodality, the medical image fusion process has the objective of improving the quality of images by decreasing error and redundancy in order to enhance overall image quality [56,57,58,59]. Clinical detection in the field of medical imaging is used for treatment and problem assessment.

In recent research trends in image fusion, motivation for analysis in image fusion is shown to have a better outcome in the latest innovations in medicine, remote sensing, and the military. With the high resolution, robustness, and effectiveness of the cost-effective image fusion technique, this methodology continues to generate critical information. Achieving the crucial data in image fusion is a more challenging and typical task because of the high cost of instruments and the huge amount of blur data present in the image. It is essential to understand the concept of image fusion. The idea of “image fusion” is the combination of two or more different or identical images to develop a new image that contains several increments of information from the various sources of images. The central aspect of image fusion is increasing the resolution of images taken from various low-resolution images. The objective is already implemented in the medical research area because coronary artery disease (CAD) is a type of disease that happens through a lack of blood supply to the heart; therefore, image transparency is required for this type of disease. The doctor also determines the report of the patient in the brain tumor disease; thus, in several modalities, applying the brain images is performed with image fusion. From the perspective of the researchers, image fusion is both exciting and challenging. Today, image fusion plays a crucial role in image classification for various applications such as satellite imaging, medical imaging, aviation, detection of a concealed weapons, multi-focus image fusion techniques, digital cameras, battle monitoring, awareness in a defense situation, the CCTV (surveillance) sector of target-tracking, gathering of intelligence concepts, authentication of the person, the geo-informatics sector, etc.

## 3. Related Work

Image fusion provides completely new competitive opportunities for technical organizations. Image fusion is a challenging technology in today’s world, and some survey results are presented below:

Li, H., et al. (1995) suitably note that the latest scheme is based on the wavelet technique and obtaining new informative images after applying IWT. The proposed scheme is much better than the Laplacian pyramid techniques in terms of effectiveness and compactness. The experimental result also includes multi-focus, SAR, infrared, set spot, and medical (MR, PET) images. It is designed with a four-part framework, but the problem is clarity. Shu-Long, Z. [2] (2002): the geometric resolution of an image is improved by the mallet algorithm presented in this study, which proposes wavelet theory to enhance the quality of a fused image. In this geometric resolution of the image, which fully depends on the high-frequency information in it, some of the algorithms do not produce a good result. Two images will be decomposed into sub-images with varying frequencies first. It is based on the wavelet transform and is entirely focused on time and frequency. Those sub-images have been converted into the resulting image with rich information after decomposition. Pradeep K. and M. Hossain [3] (2010): this study discusses the benefits of fusion in multi-modality as well as the challenges in the five critical aspects of estimation schemes, classification-based schemes, and multi-modality methods. Fusion for all rules depends on the schemes: what to fuse, when to fuse, how to fuse, and the level of fusion.

A. K., et al. [4] (2011): In this study, a new algorithm based on wavelet transform and curvelet transform is described, and the experimental results improve the lines and edges of images. The authors discussed various aspects, such as multispectral and multi-focus image fusion. The best procedure is also described in this research to achieve image fusion using wavelet transform, and the characteristics of the fused image were fully analyzed using the proposed method. Multiple features have been discussed, such as entropy, correlation coefficient, mean values, and root mean square. Sahu, V., et al. [7] (2014): Effective approaches to extracting features are proposed in this study using the transformation concept and the process of decomposition; however, this method is insufficient to find the edge information. For the various medical images, the authors used the wavelet transform. Ramandeep et al. (2014) explained various fusion techniques for medical image fusion. They defined two types of fusion methods: spatial and transform domain. They highlighted medical modalities such as MR, CT, and PET. The given concept is presented in a helpful review that shows the advantages and disadvantages of fusion techniques. It has been considered in all aspects similar to noise data, contrast, and undesired edges. Alex James and Belur [9] (2014): In this survey, the study focused on the imaging modalities in medical image fusion and the algorithms of the fusion in medical image fusion, with a final interest in the organs. Similar subjects with a large number of analogous studies and topics are combined. The practical increment and rise in medical image fusion will continue in the coming years.

Deshmukh, M.D.P., et al. [13] (2015): In this literature, the wavelet method uses remote sensing images and satellite images. The respective fused image will be more readable with the MSE and PSNR parameters. The wavelet transformation, which incorporates statistical parameters such as PSNR, SD, entropy, RMSE, and MSE, will be computed to prove this method. The authors also discussed some other methods including wavelet transform (WT), stationary wavelet transform (SWT), continuous wavelet transform (CWT), and discrete wavelet transform (DWT). Bhavana, V., et al. [14] (2015) defined this medical fusion approach as a multi-modality concept, i.e., very suitable in the medical field. In this study, gray and color images and brain images are specially used in the form of grey and color images. The authors found the edges of the images and proposed a new fusion scheme for PET and MR brain images. They also applied the wavelet transformation method to remove the distortion of the color without losing any anatomical information. Shalima, D., et al. [15] (2015) discussed spatial and temporal domain fusion techniques. Each technique has its advantages and disadvantages. They focused on the actual gap between the literature reviews. They took one single image, i.e., Lay’s image, and the image was blurred on the left side as well as the right side. Every object is focused on a single image. Fatma El-Zahraa and Mohammed Elmogy [16] (2015): The focus of this study was on the registration and fusion steps in image fusion, which are frequently debated among medical imaging modalities. Fusion procedures are described to stand up to further studies, and some of the challenges in image registration that improved the fusion techniques and medical image registration are proposed.

Tewari, K., et al. [18] (2016) presented a comparative study of image fusion techniques. They used their specific techniques to detail all information one by one, such as spatial and transform domains. They used medical images, grayscale images, and color images. It is by totally focusing on those parameters that they created a good quality picture, i.e., PSNR, MSE, entropy, etc. In this work, it has been used to compare various application images and find the suitable method for each. Li, H., et al. [19] evaluated two novel multi-focus fusion techniques on a multi-scale and multi-direction neighbor distance (MMND) scheme and classified the pixels into various mechanisms. Generally, fusion rules are applied to fuse the image; thus, they improved the performance with two schemes. All experimental work and results are validated for the proposed work, which can obtain excellent results. Liu, Z., et al. (2017) proposed a new scheme that is based on a novel fusion scheme with cartoon–texture decomposition to convert multi-focus images into cartoon content and texture content (two components). They used a fusion rule. The results show that the proposed methods provide higher quality, but the challenge is in developing a potential image decomposition algorithm to accelerate the process [22]. Nejati, M., et al. (2017) examined the new multi-focus image fusion algorithm based on a novel focus criterion. They concentrated on patterns with a few consecutive intersection points in one and two dimensions. They also used some grayscale images on a clock, leaf, Pepsi, toy, newspaper, balloon, etc. Image testing is performed with the different values. The approximate area is focused and calculated with some intersection points. Their work has been carried out on the different edges of all those images; however, the problem with this research is that the limitation of different color images has not been used, and the blurring problem is not completely discussed yet.

Xiao, D., et al. [25] (2017) analyzed the multi-focus with a robust encryption algorithm. In this paper, there is a scheme based on a security algorithm, a robust encryption algorithm based on compressive sensing. The latest schemes that may be used in data transmission volume detract and protest different attacks are discussed in this literature. Multi-focus fusion in this case is entirely based on discrete wavelet decomposition with a structurally random matrix, which reduces data volume. The authors used the same grayscale image as before. The experimental results demonstrate that the new algorithm is both effective and secure. Luo, X., et al. [26] (2017) investigated all novel multi-focus fusion results with the latest methods, i.e., higher order singular value decomposition (HOSVD) and edge intensity (EDI). HOSVD provides better image representation. Using these methods, edge intensity was presented, and HOSVD was the dominant data-driven decomposition technique. The authors took Barbara, Clock, Pepsi, and Gold Hill images for the experimental table. A further activity level measure (ALM) of the coefficient was estimated using edge intensity. Qin, X., et al. [27] (2017) developed a new multi-focus image fusion based on window empirical mode decomposition to improve the image representation. The detailed scheme was entirely focused on visual features, contrast, and local visibility. WEMD involves the image pixel as well as the grey patches. The experimental results had shown an effective scheme for capturing the detail and direction information of the source image. The disadvantage of the given research was that future work was needed to specify how to extend this for new, different color images.

Manchanda, Meenu, et al. [42] (2018) proposed a new technique for fusing medical images from many modalities using a fuzzy transform. They combined fuzzy transform pair reconstructions obtained at different times. Reconstructed error pictures produced at various stages using fuzzy transform pairs are also fused. It, too, keeps all the vital, relevant, and interconnected details of medical imaging of various modalities. Yang, Yong, et al. [43] (2018) want to combine photos with data from several sources. In this study, using structural patch decomposition (SPD) and fuzzy logic technology, we offer a new approach to multimodal medical picture fusion. To start, we use the SPD technique to obtain two important characteristics for fusion discrimination. Then, using the most important data, we build two brand new fusion decision maps—the incomplete fusion map and the supplementary fusion map. Singh, S., et al. [44] (2019) combine the deconstructed base and detail layers using a convolutional neural network (CNN) that employs consistency checking and structural patch clustering (fuzzy c-means-based). First, the brightness of each source picture is deconstructed, and then the chrominance is retrieved and separated using a color space transform. When the base layer has been broken down into its constituent parts, the next step is to utilize a CNN model that has already been trained to extract the most salient characteristics from those parts. A fusion score is calculated using an energy-based activity measure for the final feature map, and this score is then fine-tuned during consistency verification to optimize the weight map for fusing the base layers. Gambhir, D., et al. [45] (2019) investigated the wave atoms for use in a wide variety of applications, including image denoising, fingerprint recognition, and compression; it is recommended that they be used in medical picture fusion. Numerous medical imaging datasets are used to test the proposed fusion process and compare it to current best practices.

Li, X., et al. (2020) offered a new Laplacian red composition (LRD) architecture for multimodal medical picture fusion. There are two new technological features in the proposed LRD. The authors begin by outlining a Laplacian decision graph decomposition approach that incorporates picture augmentation in order to glean supplementary data, redundancy, and low-frequency subband images. Second, they established the notion of the overlapping domain (OD) and non-OD (NOD), with the OD contributing to the fusion of redundant information and the NOD being responsible for fusing complementary information due to their differences in nature. Arif, M., et al. (2020) suggested a novel approach and method of fusion for multimodal medical pictures based on the curvelet transform and the genetic algorithm. Our approach utilizes GA to maximize the features of picture fusion and clear out any doubts or haze that may have been present in the original input image. Multiple sets of medical photos have been used to evaluate the proposed approach, which is also compared to cutting-edge medical image fusion methods. Li, X. et al. [48] (2021) suggested a multimodal medical picture fusion approach that is efficient, quick, and insensitive to background noise. After decomposing an image into its structure and energy layers using a joint filter, a unique local gradient energy operator is presented for fusing the structure layer, and the abs-max rule is used to fuse the energy layer. Shehanaz, S., et al. [49] (2021) devised an optimal weighted average fusion (OWAF) for fusing medical images from different modalities to enhance the multimodal mapping performance. In our method, the multiple input modalities are decomposed using the standard discrete wavelet transform (DWT). Weights that were optimally determined using the popular particle swarm optimization technique were then applied to the resulting energy bands (PSO).

Tang, W., et al. [50] (2022) demonstrated an innovative unsupervised strategy for fusing medical pictures from different modalities using a MATR-type multiscale adaptive transformer. Instead of using plain old convolution, as in the original approach, they presented an adaptive convolution to dynamically adjust the convolutional kernel in light of the larger global complementary environment. Furthermore, an adaptive transformer is used to improve the global semantic extraction capabilities, which allows for the modelling of long-range relationships. For this reason, they have built a network with a multiscale architecture that allows us to collect relevant multimodal data at varying sizes. Alseelawi, N., et al. [51] (2022) offered a hybrid technique using NSCT and DTCWT as a viable way for fusing multimodal medical images. In the experimental investigation (PET), computed tomography, magnetic resonance imaging, and positron emission tomography were all used as input multimodality medical pictures. One strategy proposed is using a convolutional network to create a weight map that accounts for pixel-level motion data from two or more different types of multimodality medical images. Li, W., et al. [52] (2023) suggested a model that uses a combination of the CNN module and the transformer module in order to fuse many types of medical images into one. The convolutional neural network (CNN) module is used to extract texture information from images, while the transformer module is used to obtain information on the intensity distribution of pixels inside an image. Comprehensive experimental findings on the Harvard brain atlas test dataset show that the suggested technique outperforms competing methods. We propose and apply to the MR-PET and MR-SPECT multimodal medical image fusion tasks a fusion approach that maximizes local energy information and picture gradient information. Without losing the more crucial pixel distribution difference structure information of the original picture, the original image’s texture features have been retained. Zhang, C., et al. [53] (2023) presented a unique medical image fusion framework to address the shortcoming of the joint sparse model using a single vocabulary. The goal of any good fusion technique is to bring out and improve upon the already-present comparable information in the source pictures. The proposed solution decreases the amount of time spent waiting and increases the amount of time spent working.

Zhou, T. et al. [54] (2023) compile brief descriptions of many common GAN models. The article goes on to detail the benefits and uses of GAN in the medical image fusion industry. It examines the obstacles that GAN must overcome and speculates about its possible future courses of action. Liu, J. et al. [55] (2023) presented a unified fusion architecture that simply requires a basic training procedure. In this case, we alter the picture fusion process to take saliency into account. State-of-the-art fusion outcomes are attained using the suggested approach. Rajalingam, B., et al. [56] (2023) suggested and examined the hybrid multimodality medical image fusion approaches, and the key benefits and drawbacks are discussed. The quality of the resulting multimodal medical image is enhanced by using hybrid multimodal medical image fusion techniques. The experimental results of the suggested hybrid fusion approaches provide high-quality, fast-processed, and well-visualized merged multimodal medical pictures. Wang, X. et al. [57] (2023) make a case for using a transformer and a feedback mechanism to achieve multi-focus image fusion. In order to enhance the precision of focus area identification, this technique combines a transformer with a CNN and combines the local information recovered by the CNN with the global information collected by the transformer. In ref. [58], the deep label fusion (DLF) 3D end-to-end hybrid MAS and DCNN segmentation pipelines are analyzed and scored. With the multi-view attention mechanism and adaptive fusion technique in mind, the authors of [59] recommended the encoder–decoder structure of the U-Net as the core network structure upon which to build a medical image segmentation algorithm. In ref. [60], researchers provide a useful supplemental module for features by fusing them cross-wise across the CNN and transformer domains.

Xie, S., et al. [61] (2023) presented a progressive feature filter architecture in order to achieve continuous multi-modal fusion. To improve and denoise the source images, a pre-filtering module is introduced. Liu, X. et al. [62] (2023), who presented a new GAN-based approach to enhance fusion efficiency. The focus-guided discrimination technique is meant to make the target more noticeable. Alshathri, S. et al. [63] (2023) provide a professional-grade audio watermarking method using wavelet-based image fusion, Arnold transformations, and singular value decomposition for safe data transfer of medical images and reports over the Medical Internet of Things. Vasu, G.T. et al. [64] (2023) presented a multi-focus image fusion technique that makes use of a weighted anisotropic diffusion filter and a structural gradient in order to maintain the relevant edges in the final fused image. Jaganathan, S. et al. [65] (2023) offered a self-supervised 2D/3D registration approach to close the gap between domains and eradicate the requirement for paired annotated datasets that combines simulated training with unsupervised feature and pixel space domain adaptation. Li, H. et al. [66] (2023) use a Siamese conditional generator to create probabilistic local features with two different points from multi-focus images with overlapping data. Fletcher, P. et al. [67] (2023) outline the process and demonstrate the efficiency and acceptability of a new transperineal biopsy approach using electromagnetic needle tracking and a combination of magnetic resonance imaging and ultrasound, all performed under local anesthesia. AlDahoul, N. et al. [68] (2023) evaluated several RGB and depth image fusion techniques for classifying space-related objects. Thirteen fusion performance measures were used to assess the success of the studies. Bao, H. et al. [69] (2023) proposed a contextual fusion network that combines information from many scales, allowing us to simultaneously collect geographical and semantic data as well as data about the objects themselves. In order to produce the ultimate decision graph, Wu, P. et al. [70] (2023) suggested an intermediate learning algorithm and judgement module. The capacity to acquire locally relevant contextual features is strengthened. The capacity to learn locally relevant semantic information is strengthened. Wang, C. et al. [71] (2023) presented a novel fuzzy rule for uncertainty that makes use of pixel similarities using a hybrid of fuzzy set theory and deep learning. When fed feature maps recovered by the VGG-16 network, the flexible network described by Li, J. et al. [72] (2023) may dynamically optimize the necessary weights of source pictures. More texture features may be extracted from several inputs into a unified whole with the use of weight optimization in the fusing process. A model for multi-grained channel normalized fusion networks (MG-CNFNet) was proposed by Zeng, X., et al. in 2023 [73]. This model can preserve high-quality spatial texture in addition to substantial semantic information.

To make the most of the synergies between CNNs and transformers, Zheng, J. et al. [74] (2023) suggested a cross-attention and cross-scale fusion network. Yin, W. et al. [75] (2023) presented a unique adaptive visual improvement and high-significant target detection-based fusion system. The first zero-shot algorithms for multi-focus picture fusion are proposed by Hu, X. et al. [76] (2023). Deep priors of a clear and focused merged picture are effectively mined. Yang X. et al. [77] present a decoupled global–local infrared and visible image fusion transformer (DGLT-Fusion) (2023). Separating global and local knowledge acquisition into transformer and CNN sub-modules is what the DGLT-fusion does. To improve the global–local information interaction in our network, we have layered these two modules so that they mutually influence one another. In their paper, Kaya, Y. et al. [78] (2023) offer a new deep learning model for illness detection. Image fusion is used to boost the suggested model’s efficiency. The purpose of the study conducted by Zhou, H., et al. [79] (2023) was to compare the side-by-side method of assessing the ablative margin in hepatocellular carcinomas measuring 3 cm in diameter with the computed tomography image fusion method and determine which was more accurate in predicting local tumor growth. Wu, L. et al. [80] (2023) offer a fusion approach for multi-band remote sensing pictures that is built on joint representation. El-Shafai, W. et al. [81] introduced a CNN architecture for multimodal categorization of medical images in 2023. In comparison with employing pre-trained deep learning networks, the suggested network is straightforward and is directly taught using medical pictures. P. Kaur et al. [82] (2023) set out to observe how different optimization strategies performed when applied to medical imaging. As part of their routine follow-up, CT and 3D TTE were performed on 14 patients with congenital cardiac disease by Fournier, E. et al. [83] (2023). Alignment, landmarks, and superimposition are only a few of the fusion, navigation, and assessment processes that we laid out. The issue of image registration is formalized as an affine pose graph optimization, and Li, L. et al. [84] (2023) suggest fusing multiple techniques according to their uncertainties. This paves the way for a unified framework that incorporates landmarks, dense intensity registration, and learning-based methods. An unsupervised improved medical image fusion network is proposed in the article [85]. To better maintain data integrity, they used both shallow and deep restrictions. In [86], the authors suggest using an FDGNet to fuse medical images from several sources. The fusion architecture is trained with an optimal level of efficiency using a custom-created hybrid loss. The weighted fidelity loss helps keep the merged image’s brightness from dropping.

## 4. Comparative Analysis of Non-Conventional Related Work

This section compares various non-conventional multi-modal image fusion techniques in tabular form based on parameters such as methodology, merits, and demerits, as shown in Table 2.

**Table 2:** Comparison based on methodology, objectives, merits, and demerits.

Related Work	Methodology	Objectives	Merits	Demerits
Manchand, Meenu et al. [42] (2018)	Fuzzy transform	Combines many phases of an FTR pair’s reconstructed picture.	Protects all vital, useful, and interconnected data from input medical pictures of various modalities. Free from the problem of artifacts.	High computational cost.
Yang, Yong, et al. [43] (2018)	Fuzzy discrimination with structural patch decomposition	Combining images with data from many sources	Successful suppression of hue shift, leading to enhanced diagnostic performance.	Requirement of optimization to improve the computational efficiency.
Singh et al. [44] (2019)	Decomposition of hybrid layers utilizing convolutional neural networks for feature mapping and structural clustering	Improve diagnostic prediction, seeks to combine data from several sensors into a single picture.	Enhancing the structural fine details while minimizing the impact of major artefacts and noise.	Lack in pixel contrast and preservation of tiny edges.
Gambhir et al. [45] (2019)	Wave atoms transform-based medical image fusion	Medical analysis and treatment	Improved clarity and expanded information; a real advantage for faster illness diagnosis and more effective therapy.	Requirement of contrast improvement.
Li, X., et al. [46] (2020)	Laplacian redecomposition framework	Image enhancement while preserving the heterogeneous characteristics of redundant and complementary information	Qualitatively and statistically superior to other widely used fusion techniques.	Eliminates the problem of color distortion, blurring, and noise.
Arif, M., et al. [47] (2020)	Fast curvelet transform through genetic algorithm	Improve the input picture by clearing out any doubts or haze and maximizing its fusion qualities in the process.	Keeping all original data and color standards intact in the base picture, Fast computation process.	Not able to adaptively identify the breakdown level.
Li, X. et al. [48] (2021)	Joint bilateral filter and local gradient energy	To fuse the structure layer and the abs-max rule to fuse the energy layer	Easy to implementation, easy to understand and achieve high computational efficiency Has the capability to successfully used to another wide variety of image-fusion issues.	Not able to bridge the gap between multimodal medical image fusion methods and certain practical clinical applications.
Shehanaz, S., et al. [49] (2021)	Optimum weighted image fusion using particle swarm optimization	To improve the multimodal mapping performance	Powerful in both normal and noisy fusion settings in terms of information mapping, edge quality, and structural similarity.	Cannot be used in multicentral applications, has higher computational time, and experimentation is applied on normalized and registered public image database.
Tang, W., et al. [50] (2022)	Multiscale adaptive transformer	Integrate the complementing information from several modalities to improve clinical diagnosis and surgical navigation.	Constraints for preserving information at both the structural and feature levels are built using a structural loss and a region mutual information loss, and good generalization capability.	Execution time is Higher.
Alseelawi, N., et al. [51] (2022)	Hybrid approach of NSCT and DTCWT	Uses a variety of imaging modalities to compile a comprehensive picture of a disease	Highest-quality fused pictures, lower processing period, and visual quality.	In some cases, blurriness in the result is found.
Li, W., et al. [52] (2023)	Network with improved dual-branch features, trained using transformers and convolutional features	Recover texture information from images and determine the intensity distribution of pixels in a picture.	The texture details of the original image are well-kept, and the more important information about how the pixels are distributed in the original image is not lost.	Tried out a few samples of medical images.
Zhang, C., et al. [53] (2023)	Joint sparse model with coupled dictionary	To correct the flaw in the joint sparse model caused by using a single dictionary, and to emphasize and enlarge on the relevant parts of the source pictures.	Time efficiency is enhanced while less functional and structural data is lost.	Method is not implemented on color medical images as it can provide more precise treatment.
Vasu, G.T., et al. [64] (2023)	Weighted anisotropic diffusion filter	Generate a single image from many images of the same subject with different forefront and backdrop emphasis.	Efficient in edge-preserving feature.	Execution time is Higher.
Xu, H., et al. [85] (2021)	Performed surface-level and deep-level constraints in unsupervised fusion network	To preserve the unique information of source images To preserve high-quality texture details in the MRI image.	Enhanced information preservation.	High computational cost due to pixel level processing.
Zhang, G., et al. [86] (2023)	Pair feature difference guided network	To address the defects of complementary feature extraction and luminance degradation.	Preserves rich luminance in CT images, tissue texture in MRI images, and functional (PET/SPECT) details from source image.	Features that go together between source images can be taken out, less luminescence information achieved, and the network can be improved to get more features.

## 5. Experimental Results

**Figure 3:**
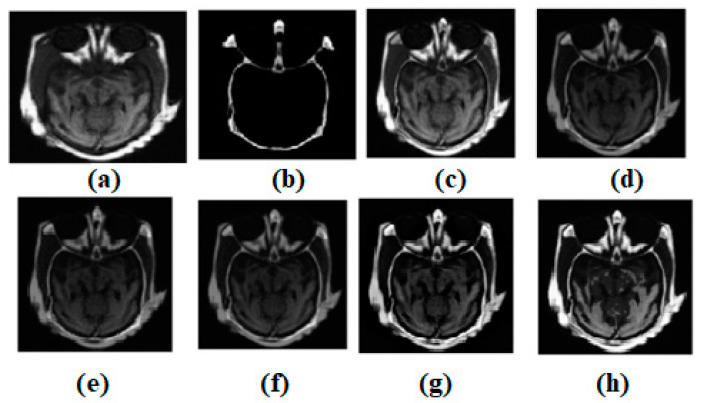
Multi-modality medical image fusion results (**a**) Source image: CT, (**b**) Source Image: MR, (**c**) Result of [44], (**d**) Result of [47], (**e**) Result of [48], (**f**) Result of [49], (**g**) Result of [51], (**h**) Result of [53].

**Figure 4:**
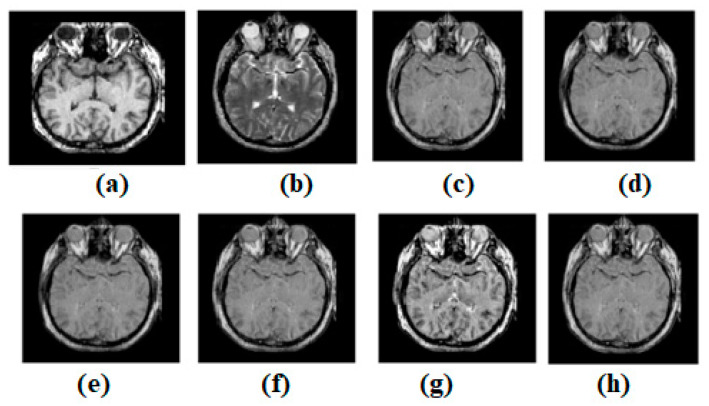
Multi-modality medical image fusion results (**a**) Source image: CT, (**b**) Source Image: MR, (**c**) Result of [44], (**d**) Result of [47], (**e**) Result of [48], (**f**) Result of [49], (**g**) Result of [51], (**h**) Result of [53].

**Figure 5:**
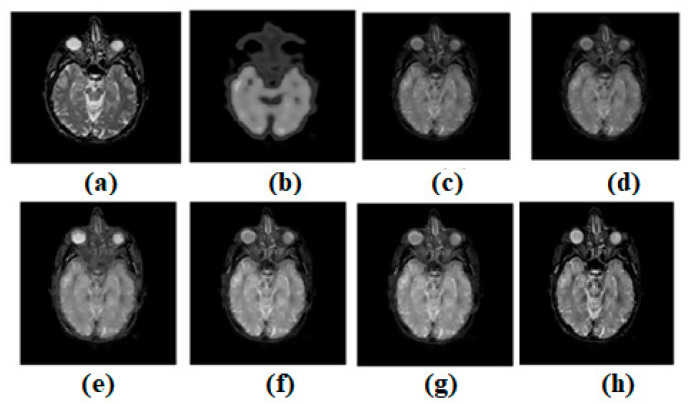
(**a**) Source image: MR-T2, (**b**) Source Image: SPECT, (**c**) Result of [44], (**d**) Result of [47], (**e**) Result of [48], (**f**) Result of [49], (**g**) Result of [51], (**h**) Result of [53].

**Figure 6:**
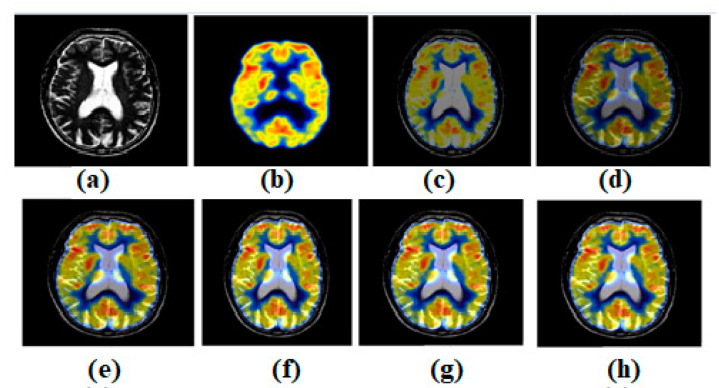
(**a**) Source image: MR, (**b**) Source Image: PET, (**c**) Result of [44], (**d**) Result of [47], (**e**) Result of [48], (**f**) Result of [49], (**g**) Result of [51], (**h**) Result of [53].

**Figure 7:**
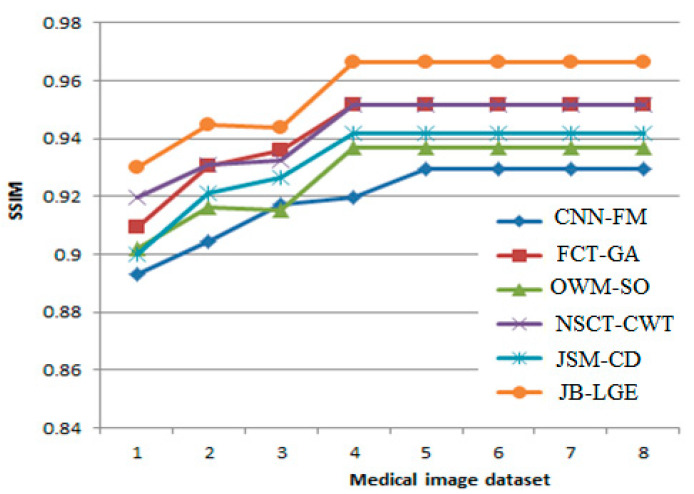
Graphical result analysis: SSIM of comparative methods between different medical image datasets over different methods- CNN-FM [44], FCT-GA [47], OWM-SO [49], NSCT-CWT [51], JSM-CD [53], JB-LGE [48].

Using the MATLAB version R2022a software, the experimental evaluation is complete. A resolution of 512 × 512 is used for the experimental results of all the images. There are numerous multi-modality effects observed in Figure 3, Figure 4, Figure 5, Figure 6 and Figure 7. Input source images are shown in Figure 3a,b. Non-conventional methods, such as [44,47,49,51,53], are combined with the established structures in all images. Figure 3c–g depicts the effects of [44,47,49,51,53], respectively. Similarly, ref. [53] different modalities are also being investigated in MR-T2 images and SPECT images. In Figure 3, the result of ref. [44] is satisfactory; however, in terms of edge preservation and texture preservation, the result of ref. [44] is not satisfactory. However, the contrasts are well-preserved. In some homogenous areas, the texture is also well-preserved. However, sharpness in the heterogeneous area is missing. In Figure 3, the result of ref. [47] is acceptable; nevertheless, when it comes to the preservation of edges and textures, the result of ref. [47] falls short of expectations. Nevertheless, the contrasts have been maintained very well. There are some areas of homogeneity, and the texture has also been nicely conserved. However, there is a lack of brightness in the heterogeneous area. In Figure 3, the outcome of ref. [49] is satisfactory; nevertheless, when it comes to the maintenance of edges and textures, the result of ref. [49] does not live up to expectations. Nevertheless, the contrasts of ref. [48] have been preserved quite beautifully. There are some places of homogeneity, and the texture has also been carefully preserved. There are also other areas where the texture has changed. The heterogeneous region, on the other hand, has an inadequate amount of light. In Figure 3, the result of ref. [51] is satisfactory; nevertheless, when it comes to the maintenance of the image’s edges and textures, the result of ref. [51] does not live up to expectations. Nevertheless, the contrasts have been maintained quite well throughout. There are certain regions of consistency, and the texture has also been maintained in an admirable fashion. On the other hand, the heterogeneous region suffers from a lack of brightness. In Figure 3, the result of ref. [53] is fine, but it falls short when it comes to keeping edges and textures. Nevertheless, the differences have been maintained in a really good way. There are some areas where everything looks the same, and the texture has also been maintained well. However, the area that is not homogenous is not very bright.

Figure 4a,b shows both the MR-T2 and SPECT images. These two images are combined into existing structures such as [44,47,49,51,53]. Figure 4c–g expresses the effects of [44,47,49,51,53], respectively. To test the most current methods, a different modality dataset is also used. As can be seen in Figure 4, the outcome of ref. [44] is acceptable, but it falls short when it comes to preserving edges and textures. However, distinctions remain clear. There are several consistent spots where the original texture has been maintained. However, there is a lack of clarity in a wildly varying domain. Figure 4 shows that the output of ref. [47] is passable, but it fails to meet expectations when it comes to edge and texture preservation. However, the contrasts are excellently preserved. Some places are consistent, and the texture has been well-preserved overall. The heterogeneous region, however, suffers from a severe shortage of illumination. While the result of ref. [49] in Figure 4 is satisfactory, it falls short when it comes to the preservation of edges and textures. The contrasts of ref. [48], however, have been retained very well. Some areas are consistent, and the original texture has been maintained with great care. The texture shifts are not limited to these spots. However, the light levels are inadequate in the area of heterogeneity. The output of ref. [51] in Figure 4 is good, but it falls short of expectations when it comes to preserving the image’s edges and textures. However, contrasts have been maintained to a satisfactory degree. Several areas of continuity have been achieved, and the texture has been preserved with great skill. In contrast, the area with uneven brightness is considered to be the heterogeneous zone. The output of ref. [53] in Figure 4 is acceptable, but it fails to maintain edges and textures. Nevertheless, the distinctions are quite well-preserved. In some spots, the uniformity of the surface has been maintained, and the texture, too, has been carefully preserved. The non-uniform region, however, has a low luminosity.

As shown in Figure 5, on the MR-T1 image and the MR-T2 image, the latest methods were also checked. Figure 5a,b shows the corresponding MR-T1 and MR-T2 images. Figure 5c–g shows the results of [44,47,49,51,53], respectively. It is evaluated from visual inspection that the approaches to visual consistency described in [53] are stronger in terms of edges and content definitions compared to the existing methods. The overall visual quality of [51,53] is comparatively better. However, [53] outperforms [51]. Refs. [44,47] also show better texture preservation; in some cases, however, artifacts are observed. Ref. [49] shows great results in terms of edge preservation and smoothness in uniform and non-uniform areas. As shown in Figure 5, ref. [44] preserves edges and textures poorly. However, differences exist. The original texture is preserved in various places. In a diverse domain, clarity is lacking. Ref. [47]’s output is adequate, but it fails to preserve the edges and texture, as shown in Figure 5. However, contrasts are maintained well. The texture is consistent in certain spots. However, the diverse zone lacks light. Ref. [49] in Figure 5 preserves edges and textures poorly. The contrasts in ref. [48] were wonderfully preserved. Some portions are consistent, and the original texture has been carefully maintained. These places are not the only ones with texture changes. In heterogeneity, light levels are inadequate. In Figure 5, ref. [51]’s result is good, but it fails to preserve the image’s edges and textures. However, contrasts have been maintained. Several areas of continuity and texture preservation have been achieved. The heterogeneous zone has inconsistent brightness. Figure 5 shows ref. [53]’s passable output, but it loses edges and textures. Nevertheless, the distinctions are well-maintained. The surface’s homogeneity and texture have been preserved in some areas. However, the non-uniform zone has poor brightness.

Input source images are shown in Figure 6a,b. All images, for non-conventional methods [44,47,49,51,53], are fused with established structures. Figure 6c–g, respectively, expresses the results of [44,47,49,51,53]. In this regard, the results of ref. [53] seem better compared to other existing methods. The result of ref. [51] is also satisfactory. However, the results of methods refs. [44,47,49] are not satisfactory. Figure 6 shows that while the result of ref. [44] is passable, it fails to adequately preserve edges and textures. Nonetheless, disparities persist. The original texture has been preserved in a few regular places. However, in a field with so many variations, clarity is lacking. As can be seen in Figure 6, while the output of ref. [47] is serviceable, it falls short of expectations in terms of maintaining edges and textures. Contrasts are kept well and the texture has been well-preserved and is uniform in some areas. Unfortunately, the heterogeneous area is severely lacking in light. As can be seen in Figure 6, the outcome of ref. [49] is passable, but it fails to adequately preserve the image’s edges and textures. However, the contrasts in ref. [48] have been preserved well. Consistency may be found in some places, and the original texture has been preserved with meticulous attention to detail. These are not the only locations where the texture has changed. However, there is not enough illumination to properly study the region’s heterogeneity. Figure 6 shows that while ref. [51] produces a good image, it fails to meet expectations in terms of edge and texture preservation. There is a high level of talent involved in maintaining the texture while also achieving continuity in a number of key locations. As opposed to this, the area of varying luminosity is known as the “heterogeneous zone.” Figure 6 shows acceptable output from ref. [53], but note that it does not preserve the edges or textures. However, there are still clear delineations between the elements. In some areas, the surface’s uniformity and texture have been preserved. However, the brightness is very low in highly textured areas.

The visual results are not sufficient to evaluate the performance of fusion methods. Hence, some popular performance metrics are utilized to evaluate the performance of multi-modality medical image fusion algorithms. Mutual information (MI), edge index (Q(ABF)), spatial frequency (SF), SSIM, and NIQE are the performance metrics. The results are evaluated and compared as shown in Table 3, Table 4 and Table 5.

For a more critical analysis of performance, the results are also evaluated on a noisy image dataset, as shown in Table 6. From Table 3, Table 4, Table 5 and Table 6, it can be seen that all methods provide better results for different performance metrics.

Additionally, a graphical result analysis is also shown in Figure 7. In Figure 5, SSIMs of comparative methods are analyzed between different medical image datasets. In most of the cases, Refs. [51,53] provide better results compared to others.

## 6. Challenges, Future Trends, and Significance

### 6.1. Current Challenges

Even though a lot of progress has been achieved in recent years, there are still some big problems to solve as regards multi-modal medical image fusion.

First, for frontier zones, making the fusion method perfect requires more work. The border regions in the source images are the transition zones between sharply defined and blurred areas, and they are often situated at sharp transitions in depth.

Second, current fusion methods rarely engage with the problem of misregistration caused by a moving body part or a medical device’s sensor shaking [87].

Third, few studies are dedicated to the development of multi-modal medical image fusion algorithms for applications in the real-time sectors of biology, medicine, industry, and practical clinical applications [88]. Most previous papers on multi-modal medical image fusion chose to use images from a natural open-access database rather than actual data from patients in the current scenario to test the efficacy of a newly proposed approach [89,90].

### 6.2. Future Prospects

In the future, researchers on this subject will be able to devote more time and energy to resolving the aforementioned issues. To begin, more complex methods for fusing medical images across boundaries will be explored [91,92]. This is an issue that cuts across all other approaches. Some first efforts have been made on this problem using approaches that mix the transform domain and the spatial domain, although the selected methodologies are quite straightforward. Second, it is envisaged that research into the misregistration problem brought on by a moving body part or medical device sensor shaking would take off [93,94]. Traditional approaches in the transform domain and the spatial domain have hit technological roadblocks while trying to solve this problem. Given their impressive propensity for learning, we believe deep learning approaches still have enough opportunity to address this issue [95,96,97]. Third, there will likely be more research conducted on multi-modal medical image fusion, with a focus on its many applications. Experiments with multi-modal medical images may be undertaken using actual patient datasets for more extensive verification than only the open access database images [98,99,100].

### 6.3. Significance

The ever-increasing volume of academic output from across the globe is making it more challenging to stay up to date in the field of multi-modal medical image fusion. Many researchers respond to this serious problem by doing a critical analysis of previously published research papers in their field and then writing a comprehensive review article on the most up-to-date advances in their research area [101]. This review paper is useful for the researcher at any stage of his investigation in medical image fusion. It helps to discover multiple research issues by evaluating the related literature. It follows that the research problem relies heavily on the literature review. The paper establishes a firm grounding in this subject matter. It finds examples of previous academic work so that new researchers may avoid duplicating efforts, properly acknowledge previous academics, and find new research directions [102]. The paper also finds the inconsistencies in the previous study, the contradictions between studies, and helps in answering the issues left unanswered by previous studies. It will also help in determining the necessity for further studies.

## 7. Conclusions

This comparative survey presents a non-conventional multimodality-based diagnostic image analysis. Human advanced data should be sensitive to improved contrast (high), pixel density and edge details, and emphasize contrast view dependence, the edges of a fusion device, and the recognition of texture. Many types of noise mistakes and improvements in the information provided in the fused image show how much data are acquired from the original image for computation. The results imply that non-conventional approaches that use the transform domain have better results when utilizing different spatial domain architectures. In addition to visual effects, the performance measurements show that approaches employing a transformed domain strategy produce better results for analogue spatial domain schemes.

## Figures and Tables

**Table 1:** Merits, demerits, and application of image fusion.

Merits	Demerits	Applications
Extracting all the useful information from the input images and merging the two images to get crucial information	In the image fusion process, noise can affect the fused image	Medical treatment
The fusion technique does not show any errors for the human preceptors	During the experimental analysis because of the application of the fusion technique	Object recognition and detection
In the process of fusion, it has robust imperfections such as misregistration and is reliable	The illumination problem in the fused images	Guidance for the navigation
The process of image fusion can show better reliability, capability, and complementary information	The processing of the data is slow when the images are fused	Surveillance for military and civilian
It is good for the identification and recognition	More input images are required for the fusion process	In the field of robotics fusion, images are mostly applied for the frequency variations in the images

**Table 3:** Performance evaluation of results using the Figure 1 input image dataset.

Method	*MI_AB,F_*	*Q_AB,F_*	*SF*	*SSIM*	*NIQE*
[44]	3.4603	0.2961	12.9609	0.9929	30.1237
[47]	3.8704	0.7537	11.1020	0.9976	22.2551
[48]	3.8104	0.7582	11.1321	0.9967	22.2321
[49]	3.8859	0.7763	11.3601	0.9976	21.6689
[51]	3.8820	0.7918	11.6428	0.9975	24.7500
[53]	3.9616	0.7974	9.6098	0.9969	23.3457

**Table 4:** Performance evaluation of results using the Figure 2 input image dataset.

Method	*MI_AB,F_*	*Q_AB,F_*	*SF*	*SSIM*	*NIQE*
[44]	3.4603	0.3387	19.9778	0.9486	22.3801
[47]	3.8704	0.3855	22.7752	0.9729	21.9882
[48]	3.8504	0.3582	21.1221	0.9667	22.0021
[49]	3.8859	0.4369	25.3019	0.9738	20.4692
[51]	3.8820	0.4276	23.1627	0.9752	21.1289
[53]	3.9616	0.3545	18.0624	0.9979	22.4254

**Table 5:** Performance evaluation of results using the Figure 3 input image dataset.

Method	*MI_AB,F_*	*Q_AB,F_*	*SF*	*SSIM*	*NIQE*
[44]	3.1319	0.5508	16.9281	0.9949	22.2935
[47]	3.2324	0.5919	14.4240	0.9883	22.1577
[48]	3.1404	0.6682	14.1421	0.9887	22.0111
[49]	3.2335	0.6449	15.1480	0.9902	22.7697
[51]	3.2482	0.6562	14.9759	0.9942	22.4171
[53]	3.2317	0.6439	15.9881	0.9996	22.1535

**Table 6:** Performance evaluation of results using Figure 3 input image dataset with Gaussian noise.

Method	*MI_AB,F_*	*Q_AB,F_*	*SF*	*SSIM*	*NIQE*
[44]	3.0889	0.3118	41.2059	0.9893	22.7915
[47]	3.1979	0.2905	38.9116	0.9905	21.7460
[48]	3.1204	0.3562	38.1021	0.9917	21.7321
[49]	3.1979	0.3505	42.4170	0.9908	20.5352
[51]	3.1990	0.3591	39.8955	0.9938	22.2407
[53]	3.1980	0.3618	36.5719	0.9995	19.5832

## Data Availability

Not applicable.
